# Exome sequences versus sequential gene testing in the UK highly specialised Service for Limb Girdle Muscular Dystrophy

**DOI:** 10.1186/s13023-017-0699-9

**Published:** 2017-09-06

**Authors:** Elizabeth Harris, Ana Topf, Rita Barresi, Judith Hudson, Helen Powell, James Tellez, Debbie Hicks, Anna Porter, Marta Bertoli, Teresinha Evangelista, Chiara Marini-Betollo, Ólafur Magnússon, Monkol Lek, Daniel MacArthur, Kate Bushby, Hanns Lochmüller, Volker Straub

**Affiliations:** 1 The John Walton Muscular Dystrophy Research Centre, Institute of Genetic Medicine, Central Parkway, Newcastle upon Tyne, NE1 3BZ UK; 2 Muscle Immunoanalysis Unit, Newcastle upon Tyne Hospitals NHS Foundation Trust, Newcastle upon Tyne, NE2 4AZ UK; 3 Northern Genetics Service, Institute of Genetic Medicine, Newcastle University, Newcastle upon Tyne, UK; 4 Northern Institute for Cancer Research, Newcastle University, Newcastle upon Tyne, UK; 5 deCODE Genetics, Reykjavik, Iceland; 6 Analytic and Translational Genetics Unit, Massachusetts General Hospital, Boston, USA; 7 Newcastle University John Walton Muscular Dystrophy Research Centre, Institute of Genetic Medicine, Newcastle upon Tyne, UK

**Keywords:** Myopathy, Limb girdle muscular dystrophy, Exome, Titinopathy, Collagen VI related dystrophy, Mosaicism

## Abstract

**Background:**

Limb girdle muscular dystrophies are a group of rare and genetically heterogeneous diseases that share proximal weakness as a common feature; however they are often lacking very specific phenotypic features to allow an accurate differential diagnosis based on the clinical signs only, limiting the diagnostic rate using phenotype driven genetic testing. Next generation sequencing provides an opportunity to obtain molecular diagnoses for undiagnosed patients, as well as identifying novel genetic causes of muscle diseases. We performed whole exome sequencing (WES) on 104 affected individuals from 75 families in who standard gene by gene testing had not yielded a diagnosis. For comparison we also evaluated the diagnostic rate using sequential gene by gene testing for 91 affected individuals from 84 families over a 2 year period.

**Results:**

Patients selected for WES had undergone more extensive prior testing than those undergoing standard genetic testing and on average had had 8 genes screened already. In this extensively investigated cohort WES identified the genetic diagnosis in 28 families (28/75, 37%), including the identification of the novel gene *ZAK* and two unpublished genes. WES of a single affected individual with sporadic disease yielded a diagnosis in 13/38 (34%) of cases. In comparison, conventional gene by gene testing provided a genetic diagnosis in 28/84 (33%) families. Titinopathies and collagen VI related dystrophy were the most frequent diagnoses made by WES. Reasons why mutations in known genes were not identified previously included atypical phenotypes, reassignment of pathogenicity of variants, and in one individual mosaicism for a *COL6A1* mutation which was undetected by prior direct sequencing.

**Conclusion:**

WES was able to overcome many limitations of standard testing and achieved a higher rate of diagnosis than standard testing even in this cohort of extensively investigated patients. Earlier application of WES is therefore likely to yield an even higher diagnostic rate. We obtained a high diagnosis rate in simplex cases and therefore such individuals should be included in exome or genome sequencing projects. Disease due to somatic mosaicism may be increasingly recognised due to the increased sensitivity of next generation sequencing techniques to detect low level mosaicism.

**Electronic supplementary material:**

The online version of this article (10.1186/s13023-017-0699-9) contains supplementary material, which is available to authorized users.

## Background

Limb Girdle Muscular Dystrophies (LGMDs) are a clinically and genetically heterogeneous group of more than 30 rare disorders which frequently overlap in their genetic aetiology and clinical presentations with other myopathies [1]. Current diagnostic algorithms for LGMDs include clinical assessment, muscle biopsy and MRI to direct sequential gene by gene testing [2–4]. However, as in other rare diseases [5], there is frequently a delay to diagnosis in LGMDs and other myopathies due to the large number of genetically distinct diseases [6–8]. Obtaining a genetic diagnosis is crucial for an individual affected by a rare disease in order to optimise clinical care, enable accurate genetic counselling as well as curtailing the ‘diagnostic odyssey’ which may include costly and invasive investigations, inappropriate treatment, and psychological distress [5, 9–11]. In addition a genetic diagnosis enables access to natural history studies and interventional clinical trials, which are increasing and are becoming a concrete opportunity for some of the LGMDs [12].

Next generation sequencing techniques (NGS) provide a potential way to overcome diagnostic delays due to genetic heterogeneity and also the possibility to identify novel genetic causes of muscle disorders [13, 14]. Several studies have reported on the application of gene panels [15–23] or WES [24, 25] for the diagnosis in undiagnosed muscle disease, achieving diagnostic rates from 16 to 76%, with the highest diagnostic rate achieved in patients with no prior genetic testing [26].

In the UK, Highly Specialised Services for rare diseases are commissioned on a national level, and for LGMDs this service (referred to in this study as the UK LGMD clinic) is based in Newcastle upon Tyne. In this diagnostic advisory service patients with LGMD and related phenotypes from all over the UK are assessed. The same clinical centre also provides a clinical service to local patients with genetic neuromuscular diseases in the North of England [27]. In this population a genetic diagnosis is achieved in 63% of individuals with an LGMD phenotype, meaning that a third of patients with LGMD remain undiagnosed [27, 28].

In this study, we performed WES in a cohort of patients with presenting with limb girdle weakness who had been evaluated at our centre and in whom extensive investigations, often taking place over a decade or more, had not been able to reach a precise diagnosis. We also evaluated the status quo in terms of genetic testing for referrals to the UK LGMD clinic in a 2 year period. Our aims were firstly, to identify genetic aetiology of disease, including variants in known and novel disease genes, in a cohort of previously extensively investigated myopathy patients, and secondly to consider the clinical utility of WES as a diagnostic tool in a clinical setting by comparison to diagnostic outcomes using standard gene by gene testing.

## Results

WES was performed in 135 individuals (104 affected, 31 unaffected relatives) from 75 families who had been assessed in the UK LGMD clinic in the past and remained undiagnosed. Ninety-one affected individuals from 84 families were investigated using standard genetic testing procedures at the UK LGMD clinic in the 24 month period examined.

### Patients selected for WES had been more extensively investigated than those undergoing standard testing

**Fig. 1:**
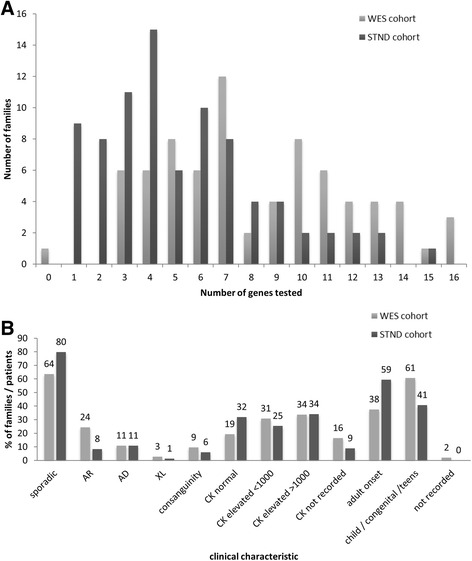
Clinical characteristics and prior testing in the cohorts selected for WES and those undergoing standard testing. **a** - The number of individual genes screened by any method per family in WES cohort or standard testing cohorts. **b** - Comparison of inheritance pattern, serum CK and age of disease onset in cohort selected for WES or undergoing standard testing. Numbers on columns indicate the percentage of patients or, for inheritance pattern, families with a characteristic

Patients selected for WES had undergone more extensive prior investigations than the cohort attending the UK-LGMD clinic (Additional file 1: Tables S1 and Additional file 2: Table S2): muscle biopsy was performed in at least one individual in 74/84 (88%) of UK LGMD-clinic families, compared to 74/75 (99%) of WES families; and the mean number of individual genes tested per family was 3 for those undergoing standard testing, compared to 8 genes previously tested in the cohort selected for WES (Fig. 1a).

We compared clinical characteristics which may impact on rate of diagnosis, including the inheritance pattern, serum creatine kinase (CK) levels and age of symptom onset, between the standard testing and WES cohort (Fig. 1b). Both cohorts mainly comprised patients with sporadically occurring disease; the WES cohort had a higher proportion of families with an autosomal recessive inheritance pattern (24% compared to 8% in the standard testing cohort). Similar proportions of patients had dominant or X-linked inheritance patterns, and rates of consanguinity were similar between groups (Fig. 1). The majority of patients in both the WES and standard testing cohorts had an elevated CK level (59% in standard testing and 65% in WES cohort). A greater proportion of patients in the standard testing cohort had adult onset of symptoms (59%, compared to 38% in the WES cohort). In the combined WES and standard testing cohorts the five most frequently tested genes were sequencing of *ANO5*, *CAPN3, FKRP, LMNA* and sequencing of *TTN* exons 293 and 308, which are mutation hotspots for Hereditary Myopathy with Early Respiratory Failure, a relatively frequent diagnosis in the North of England [29].

### A genetic diagnosis was obtained for 37% of patients using WES and for 33% patients by standard testing

**Fig. 2:**
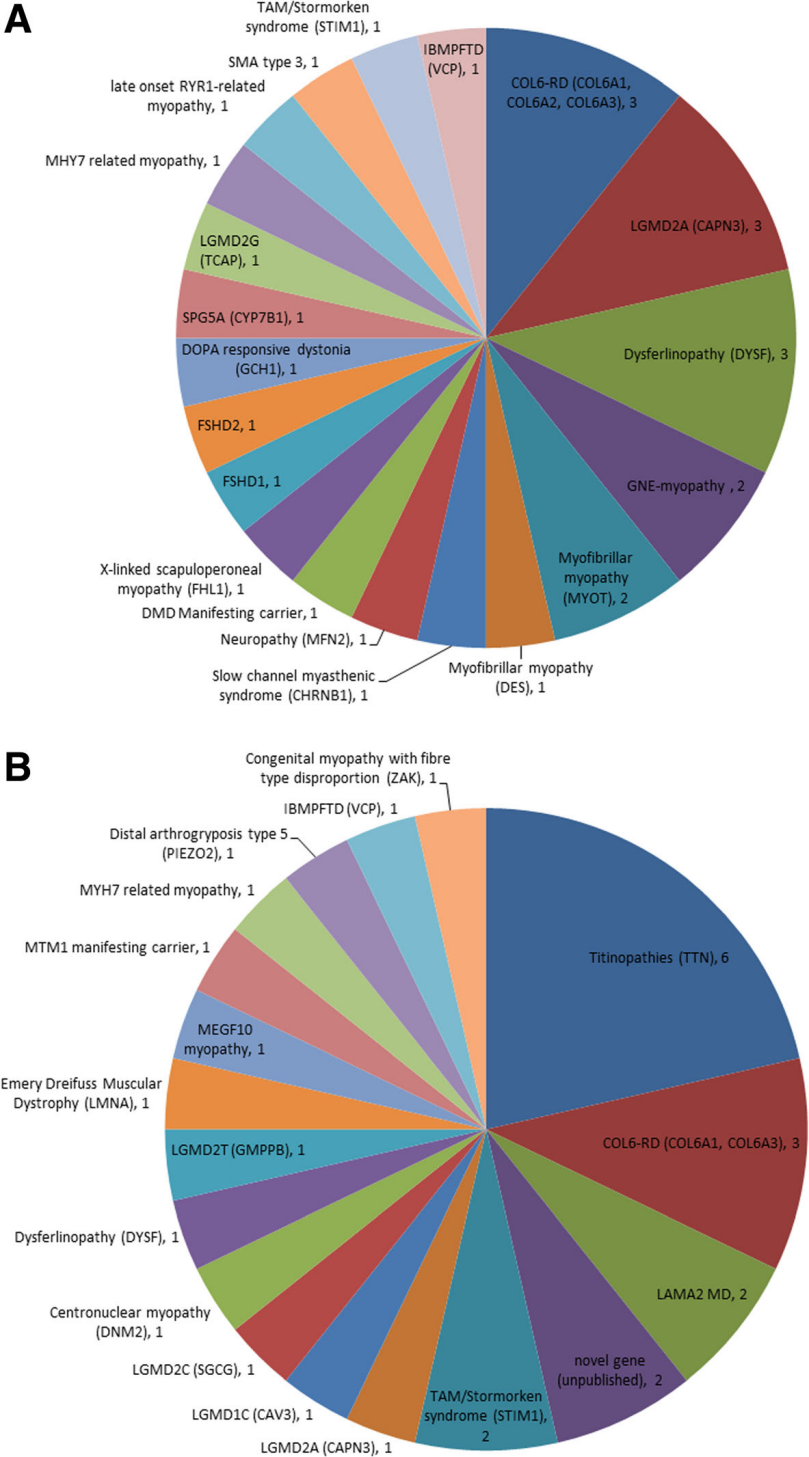
Diagnoses made by standard testing (**a**) and by WES (**b**). COL6-RD – Collagen VI related dystrophy, *LAMA2* MD –*LAMA2* related muscular dystrophy, TAM – Tubular Aggregate Myopathy, IBMPFTD – Inclusion Body Myopathy with Pagets disease and Frontotemporal Dementia

In the WES cohort a genetic diagnosis due to mutations in a known disease gene were made in 25/75 of families (Fig. 2), and disease due to mutation(s) in a novel disease genes (*ZAK* [30], and two currently unpublished genes) was made in a further 6 individuals from 3 families (3/75), making the overall rate of diagnosis 28/75 (37%). Using standard genetic testing a diagnosis was obtained for 28/84 (33%) families (Fig. 2). Fifteen panel tests were requested in 14 families in the standard testing cohort and one panel test was request in a family in the WES cohort (details of panel tests are included in Additional file 3). As a result of panel testing a single family was diagnosed with a peripheral neuropathy due to a *MFN2* mutation (Additional file 1: Table S1).

A specific family structure was not required for an individual to be selected for WES, and the majority of patients included were single individuals with no family history or additional family members available for sequencing. The diagnostic rate in sporadically occurring disease was 32% (15/47 families), and where only index patients’ DNA was available was 34% (13/38 families). The highest diagnostic rate was obtained in families with an autosomal dominant history, where 6 out of 8 families were diagnosed (75%). In families with an autosomal recessive inheritance pattern 5 of 17 families were diagnosed (29%). In the nine families where trio WES data was obtained a diagnosis was obtained in two families (22%).

### Genetic diagnoses made by standard testing

Diagnoses made by standard genetic testing are shown in Fig. 1a, and include LGMDs as well as diseases with overlapping phenotypes, such as collagen VI-related dystrophy (COL6-RD) and myofibrillar myopathies. Notably diagnoses in three families, of fascioscapulohumeral muscular dystrophy (FSHD) types 1 and 2 and Duchenne manifesting carrier due to an intragenic deletion, would have been missed by WES given the limitations of WES currently for detecting copy number changes or more complex genetic abnormalities as in FSHD.

### Genetic diagnoses made by WES

Disease causing mutations in 17 previously known muscle disease genes were identified by WES (Fig. 2b). Additional clinical investigations to evaluate the plausibility of a diagnosis suggested by WES findings, so-called ‘reverse phenotyping’, were required in most instances to confirm the diagnosis indicated by WES (Table 1). The most frequent genetic diagnosis made by WES was disease due to mutations in *TTN,* as this large gene was not routinely sequenced in the pre-NGS era. Titinopathy phenotypes encompass a wide spectrum and in this study distal myopathy (family WES5), congenital myopathy (families WES18 and WES23), and LGMD (families WES19, WES29, WES61 – reported in detail elsewhere [31]).

**Table 1:** Confirmed diagnoses made by exome sequencing

ID	gene	c.DNA change*	Protein change	Reported	Segregation studies / Reverse phenotyping investigations
5	*TTN*	c.107840 T > A	p.(Ile35947Thr)	reported	Present in 3 affected family members / muscle MRI and MB re-analysis: findings consistent with titinopathy
9	*COL6A1*	c.957G > T	p.(Lys319Asn)	reported	De novo / Repeated Sanger sequencing in DNA extracted from blood and from cultured fibroblasts demonstrates presence of mutation at low level consistent with mosaicism – see text and Fig. 3
13	*PIEZO2*	c.2136C > A	p.(Met712Ile)	novel	Present in 4 affected family members / Phenotype consistent with Distal Arthrogryposis Type 5
18	*TTN*	c.48312 + 2_48,312 + 15del	ExSS	novel	De novo ExSS; maternally inherited nonsense / MB re-analysis consistent with titinopathy
		c.1933G > T	p.(Glu645^a^)	novel	
19	*TTN*	c.107377 + 1G > A	ESS	reported	Variants inherited in *trans* / MB re-analysis consistent with titinopathy^b^
		c.97863G > A	p.(Trp32621^a^)	novel	
23	*TTN*	c.50170C > T	p.(Arg16724^a^)	novel novel	Both variants present in two affected siblings, and confirmed in *trans* / MB re-analysis consistent with titinopathy
		c.19091G > A	p.(Cys6364Tyr)		
25	*GMPPB*	c.860G > A	p.(Arg287Gln) p.(Cys113Tyr)	reported novel	Segregation not possible / MB consistent with dystroglycanopathy
		c.338G > A			
26	*LAMA2*	c.611C > T	p.(Ser204Phe)	novel novel	Both variants present in 2 affected siblings, parental DNA not available / MRI brain demonstrated white matter changes, skin biopsy immunoanalysis demonstrated laminin α2 partial absence (previously reported [57])
		c.4533delT	p.(Gly1512Alafs^a^83)		
	*TTN*	c.107377 + 1G > A	ESS	reported novel	Both variants present in 2 affected siblings and confirmed in *trans* / MB re-analysis consistent with titinopathy^b^
29					
		c.98603delT	p.(Phe32868Serfs^a^11)		
30	*MYH7*				Present in 2 affected family members and one with non-penetrance / MB reassessment
		c.5533 N > T	p.(Arg1845Trp)	reported	
32	*LAMA2*	c.6992 + 5G > A	ExSS	novel	c.2049_2050delAG maternally inherited, paternal DNA not available / MRI brain demonstrated white matter changes, skin biopsy immunoanalysis demonstrated laminin α2 partial absence
		c.2049_2050delAG	p.(Arg683fs)	novel	
	*MEGF10*	c.2049_2050delAG	p.(Arg683Serfs^a^21)	reported	Segregation in unaffected siblings consistent with AR inheritance in *trans* / MB re-analysis, muscle MRI
36		c.352 T > C p.Cys118Arg	p.(Cys118Arg)	novel	
		c.1426 + 1G > T	ESS	novel	Segregation in unaffected siblings consistent with AR inheritance / *CAPN3* sequenced in prior testing: at that time c.1746-20C > G was classified as a benign polymorphism and c.759_761delGAA was not detected by Sanger sequencing
39	*CAPN3*	c.759_761delGAA c.1746-20C > G	p.(Lys254del)	reported	
			ExSS	reported	
47	*COL6A1*	c.362A > G	p.(Lys121Arg)	reported	Present in 5 affected family members / muscle MRI consistent with COL6-RD
49	*VCP*	c.329 N > A	p.(Arg110His)	reported	Present in 4 affected family members / no additional investigations required
52	*COL6A3*	c.6265G > C	p.(Gly2089Arg)	novel	Present in 2 affected family members / no additional investigations required
56	*STIM1*	c.242G > A	p.(Gly81Asp)	reported	De novo / MB re-analysis, USS abdomen, biochemistry and haematology parameters assessment identified abnormalities consistent with *STIM1* mutation (reported separately [58])
57	*LMNA*	c.746G > A	p.(Arg249Gln)	reported	De novo / no additional investigations required
59	*DNM2*	c.1684_1686delAAG	p.(Lys562del)	reported	De novo / MB review
61	*TTN*	c.107377 + 1G > A	ESS	reported	Maternally inherited nonsense, paternal DNA not available / CT of lower limb muscles and phenotype review^b^
		c.87529A > T	p.(Lys29177^a^)	novel	
62	*STIM1*	c.262A > G	p.(Ser88Gly)	novel	De novo / MB re-analysis, USS abdomen, biochemistry and haematology parameters assessment identified abnormalities consistent with *STIM1* mutation (reported separately [58])
65	*CAV3*	c.136G > A	p.(Ala46Thr)	reported	Present in two affected family members / Additional immunoanalysis of muscle biopsy demonstrated absence of caveolin 3
67	*MTM1*	c.1054-2_1054-1delinsTT	ESS	novel	Segregation not possible / X-inactivation studies demonstrated skewed X-inactivation, muscle MRI and MB review consistent with *MTM1* manifesting carrier phenotype
71	*DYSF*	c.895 N > C	p.(Gly299Arg)	reported	Consistent with AR inheritance in *trans* / MB review and repeat immunoanalysis
		c.2875C > T	p.(Arg959Trp)	reported	
75	*SGCG*	c.787G > A (Hom)	p.(Glu263Lys)	reported	Homozygous in 2 affected siblings and heterozygous in parents and unaffected sibling / Variant was detected by prior testing but classified as of uncertain clinical significance. Muscle biopsy was of inadequate quality to perform immunoanalysis but phenotype in accordance with this diagnosis

^a^All reported variants are heterozygous except where indicated as Hom – homozygous

^b^Three families with a shared phenotype and mutation in *TTN* have been reported separately [30]. ESS – essential splice site; ExSS – extended spice site; MB re-analysis –including additional relevant Immunoanalysis; MB review – no additional immunoanalysis performed. Reference sequences: *CAPN3* ENST00000397163; *CAV3* ENST00000343849; *COL6A1* ENST00000361866; *COL6A3* ENST00000295550; *DNM2* ENST00000389253; *DYSF* ENST00000258104; *GMPPB* ENST00000308375; *LAMA2* ENST00000421865; *LMNA* ENST00000368300; *MEGF10* ENST00000508365; *MTM1* ENST00000370396; *MYH7* ENST00000355349; *PIEZO2* ENST00000580640; *SCN9A* ENST00000409672; *SLC2A1* ENST00000426263; *STIM1* ENST00000300737; *TTN* ENST00000589042; *VCP* ENST00000417448

Explanations for why the genetic diagnoses made by WES had been missed by standard diagnostic pathways are summarised in Table 2. In five instances mutations in known genes were identified in patients with typical phenotypes where this particular genetic test had not yet been requested due to the number of candidate diagnoses associated with a non-specific phenotype. At the time of study *GMPPB* and *STIM1* were recently identified myopathy genes and therefore their testing was not yet part of routine diagnostic screening. Diagnosis in an *MTM1* manifesting carrier (WES67) and a family with distal arthrogryposis due to *PIEZO2* mutation (WES13) were missed due to the rarity of these disorders [32–34], which meant that the necessary genes had not been selected for screening by standard testing.

**Table 2:** Reason for disease causing mutation was not identified by standard testing. The reason “Genetic heterogeneity” was selected when the presenting phenotype was consistent with that reported due to mutations in this gene but had not been previously tested as several genes are associated with this phenotype

Reason	No. of occurrences	Genetic diagnoses
Whole gene sequencing not previously available	6	*TTN*
Genetic heterogeneity	5	*COL6A1, COL6A3, VCP, DNM2, SLC2A1*
Recent gene discovery	3	*STIM1, GMPPB*
Specific muscle biopsy immunoanalysis not originally performed	3	*LAMA2* (2 occurrences), *CAV3*
Polymorphism reclassified as pathogenic	3	*SGCG, CAPN3 (*2 occurrences)
Mutation missed by Sanger sequencing	2	*CAPN3, COL6A1* mosaic
Disease rarity	2	*PIEZO2, MTM1 manifesting carrier*
Atypical phenotype	2	*MEGF10, DYSF*
Inheritance pattern misleading	1	*MYH7*
Mutation missed by previous gene screening technique (DHPLC)	1	*LMNA*

In seven instances the explanations for why diagnosis due to mutation(s) in known genes had not previously been made were related to the fact that these patients had been undiagnosed for several years, including muscle biopsy tissue being lost or unsuitable for further analysis or biopsy being performed before immunohistochemistry that is now routine was available; reclassification of polymorphisms as pathogenic; and historical screening of a gene (*LMNA*) using DHPLC rather than genetic sequencing. On two occasions mutations were missed by diagnostic Sanger sequencing. These findings indicate the need to revisit strong candidate genes.

Several patients had not been diagnosed due to the limitations of phenotype-driven testing. These include an individual with normal dysferlin expression on immunoanalysis who was compound heterozygous for two missense mutations in dysferlin, a rarely reported phenotype which may become more common as genetic testing may precede muscle biopsy [8, 35]. Similarly one patient with adult onset symptoms had compound heterozygous mutations in a congenital myopathy gene, *MEGF10* (WES36), who would not have been diagnosed by phenotype driven testing as diagnosis in this individual expands the phenotype associated with mutations in this gene. In one family (WES30) the apparent inheritance pattern, with an affected male proband, affected maternal uncle and unaffected mother, had directed testing to X-linked genes, but in fact the phenotype was compatible with autosomal dominant disease due to *MYH7* mutation as identified on WES with non-penetrance in the mother.

### Mosaicism in COL6A1 detected by WES

**Fig. 3:**
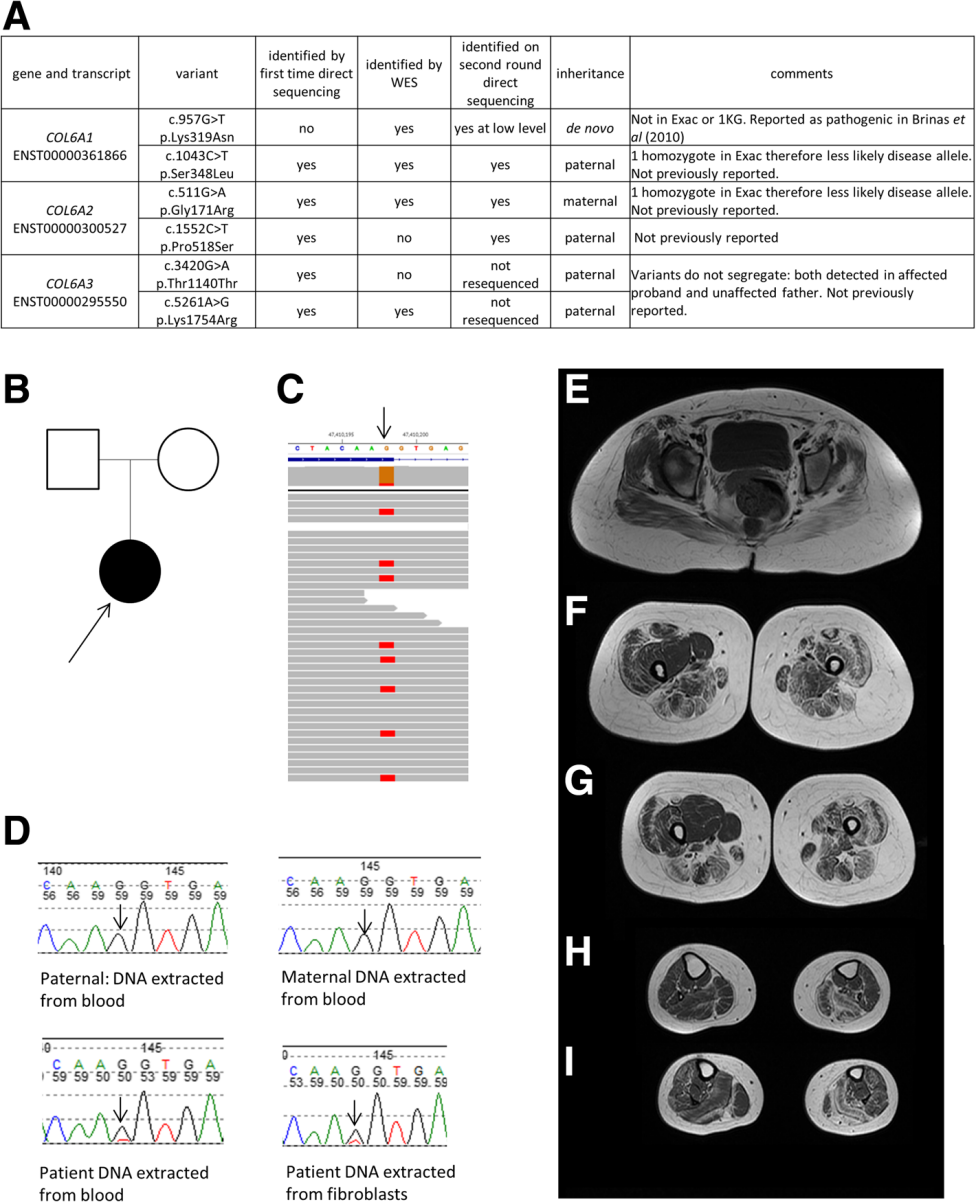
Sequencing and muscle MRI in Patient WES9. **a** – variants in collagen VI genes identified by Sanger sequencing and WES in this patient. **b** – Pedigree. **c** – Visualisation of exome sequencing reads in patient DNA from blood with arrow indicating chr21:47,410,198 G > T (hg19) corresponding to *COL6A1* c.957G > T; **d** – Sequencing chromatograms in patient DNA and parental DNA with *COL6A1* c.957G > T variant indicated by arrows, height of sequencing peak in patient with DNA extracted from both blood and fibroblasts is lower than expected suggestive of mosaicism. Fibroblasts were obtained from skin biopsy taken from the right arm, which clinically is less severely affected than the left. **e**-**i** – T1 weighted axial MRI images of pelvis and lower limbs. In the pelvis (E) there is diffuse involvement of the gluteus maximus and medium with the left side more severely affected. In the thighs (F&G) there is sparing of gracilis and vastus medialis on the right, and diffuse involvement of all muscles on the left. There is central sparing of the vastus lateralis in the thighs and a ‘central shadow’ of increased signal intensity in the rectus femoris on the left (F&G) and less marked on the right, both of which are typical of COL6-RD [56]. In the calves there is peripheral involvement of the gastrocnemius and soleus on the left, with less pronounced peripheral involvement of the distal gastrocnemius on the right

The second most frequent diagnosis made by WES and also frequently made in the standard testing cohort (Fig. 2) was collagen VI-related dystrophy (COL6-RD). Patient WES9 had a typical COL6-RD phenotype, and had had prior diagnostic Sanger sequencing of *COL6A1, COL6A2* and *COL6A3* which identified five variants of uncertain clinical significance (Fig. 3). We observed a discrepancy in detection of variants in these genes by Sanger sequencing and by WES (Fig. 3a). Following repeated Sanger sequencing of variants detected by WES we noted that a *COL6A1* variant (c.957G > T p.Lys319Asn) detected by WES but not by initial Sanger sequencing was present at a low level in DNA from patient blood and cultured fibroblasts but not in DNA from either parent, consistent with a de novo mosaic mutation (Fig. 3d). One patient with this mutation has been previously reported [36] with a more severe phenotype (loss of ambulation aged 7 years, forced vital capacity (FVC) of 15% and non-invasively ventilated aged 11 years) than our patient who is now 25 years old and remains ambulant with an FVC of 56% at last assessment. There was evidence against the pathogenicity of the other identified variants in *COL6A1*, *COL6A2* and *COL6A3* (Fig. 3). Given that mosaicism for collagen VI genes is a recently recognised cause of disease [37] we feel that the de novo mosaic *COL6A1* variant is likely to be the cause of disease in our patient. We observed asymmetry in the pattern of muscle involvement on muscle MRI (Fig. 3e) and on clinical assessment, which is atypical for COL6-RD and could be explained by somatic mosaicism.

## Discussion

We performed WES in 75 families presenting with genetically undiagnosed limb girdle weakness and compared this to phenotype driven genetic testing in a cohort of patients investigated in the UK LGMD clinic over a 2 year period. Our results show that, firstly, patients with undiagnosed myopathies often undergo a long and costly diagnostic odyssey; and secondly that WES was able to outperform standard genetic testing, even in a cohort of patients who had undergone more extensive prior testing. Sequential gene by gene testing is time consuming, with individual tests often taking several months. In the standard testing cohort 67% of patients remained undiagnosed 2 years after attending the UK LGMD clinic, and many of the patients selected for WES had been symptomatic for more than 10 years. In this time accurate information about prognosis, optimal medical management or genetic counselling was unavailable.

Precisely where in the diagnostic pathway WES should be undertaken for optimum benefit, has not yet been defined, although earlier application is likely to substantially improve the diagnostic rate [19], as is probable for the diagnosis of other genetic diseases by WES [9]. Notably, patients selected for WES were further into the diagnostic journey than those attending the UK LGMD clinic, with on average 8 genes tested per family in the WES cohort in comparison to 3 genes in the standard testing cohort, and it is probable that earlier application would have increased our diagnostic rate by WES further. Given the rapid pace of change in diagnostic genetic testing we do not propose a new diagnostic pathway at this point, but recommend that any new NGS based pathway for diagnosis of LGMDs incorporates assessment by a neuromuscular specialist clinician to ensure appropriate screening for disease not likely to be detected by NGS which may also present with proximal weakness, such as FSHD or dystrophinopathies, is performed in relevant cases.

Several studies have used a gene panel, rather than WES or genome sequencing, approach for diagnosis in neuromuscular disease [15–23], and whether this is best practice in diagnostics is a topic for debate [13]. Although gene panels may be more cost effective in terms of cost per gene sequenced than individual genetic tests, and also have the advantage of greater depth of coverage in comparison to WES, they are still phenotype-driven testing and as such require the correct panel of genes to be selected [13, 38] which may limit the rate of diagnosis. In this study panel tests were performed in 15 families (Additional file 1: Tables S1 and Additional file 2: Table S2), and only in one instance did this identify the cause of disease. The wide range of genetic diagnoses made in patients who had undergone WES in this study illustrates the overlap in phenotypes between several neuromuscular disorders which is problematic for selecting a specific gene panel. For example patient WES32 presented with adult onset proximal weakness with CK >1000 iu/L and was therefore considered to have an LGMD, but actually had disease due to *LAMA2* mutations (Additional file 1: Table S1), more usually associated with congenital muscular dystrophy. Similarly, patient WES31 and WES67 had adult onset disease due to mutations in genes typically associated with congenital myopathies (*MEGF10* and *MTM1* respectively; Additional file 1: Table S1). Restricting testing to a narrow phenotype-determined panel of genes may therefore miss atypical phenotypes or genes with a wide range of associated phenotypes.

Patients who remain undiagnosed currently may represent novel genetic diseases, and obtaining WES data, in addition to allowing for a large number of genes to be selected for an initial ‘panel by exome’ approach, then allows for patients with no likely pathogenic variants in relevant disease genes to be promptly transitioned to research for further analysis, as occurred in this cohort with some success, or into international data sharing projects to facilitate new disease gene identification [39]. It is also conceivable that some undiagnosed patients may have acquired forms of muscle disease, for example immune-mediated necrotizing myopathies including those due to anti-HMGCR and anti-SRP antibodies, which are diagnoses with therapeutic implications [40]. Alternatively those remaining undiagnosed may have intronic mutations not detected by WES. Indeed, the recent identification of a deep intronic *COL6A1* mutation in 25% of individuals with a COL6-RD phenotype in who initial testing for *COL6A1, COL6A2* and *COL6A3* mutations was negative [41], suggests that such mutations have the potential to account for many patients undiagnosed by WES.

Explanations for why pathogenic mutations in known genes were identified by WES but not by standard testing are relevant to many rare diseases, and include the dynamic nature of assignment of pathogenicity status, an issue which is not restricted to variants identified by WES, and single gene diagnostic testing being unable to keep pace with the rapid rate of new disease gene discovery and resultant increasing genetic heterogeneity (Table 2). The individual with a mosaic *COL6A1* mutation highlights the ability of next generation sequencing techniques to identify somatic mosaicism at a lower level that Sanger sequencing. As next generation sequencing becomes more prevalent this may be an increasingly recognised contributor to human disease [42–44].

The high proportion of patients diagnosed with pathogenic *TTN* mutations in the WES cohort is because it is only with the advent of NGS that is has been possible to sequence this very large gene. Titinopathies may account for a large share of as yet undiagnosed muscle disease [45] although whether this finding may be restricted to northern Europe for example, or be globally true remains to be shown. It is therefore likely that some patients in the standard testing cohort in this study who remain undiagnosed may have pathogenic *TTN* variants. In addition to those with a diagnosis of titinopathy reported here, potentially damaging *TTN* variants were observed in other patients in whom we did not feel there was sufficient evidence to define them as pathogenic. The difficulty of determining pathogenicity of titin variants is likely to be limiting the diagnosis of titinopathy to those who fulfil conservative diagnostic criteria, therefore this diagnosis may therefore be underreported even in patients where TTN has been sequenced.

Given the high frequency of rare and truncating titin variants in the general population functional confirmation of pathogenicity of titin variants should be undertaken [46], as was performed and reported separately for families WES19, WES29 and WES61 [31]. Such specialised analysis is not routinely available for all patients with truncating titin variants within the service provided by the UK Highly Specialised Service (HSS) for LGMD. Nonetheless we felt that following multidisciplinary evaluation of genetic and segregation data, clinical features, muscle MRI and muscle biopsy histopathology and immunoanalysis (Additional file 3: Fig. S1) that affected individuals in families WES18 and WES23, in who truncating titin variants were also identified, were most likely affected by titinopathies. It would be prudent to continue to review assessments of pathogenicity of *TTN* variants as our understanding of the complexities of this gene improves. Critical to this are collaborative efforts to share data and define a strategy for accurate interpretation [46, 47].

WES in this study was performed on a research basis and therefore with no specific cost for comparison, but in general WES rates vary by sequencing provider and according to the degree of interpretation. The real cost in terms of time taken to interpret, confirm and segregate variants as well as clinical assessment +/−additional investigations to assess plausibility of a diagnosis suggested by WES is however not currently quantified, and there is concern that these costs may be substantial [48]. Reverse phenotyping investigations, such as additional muscle biopsy immunoanalysis or muscle MRI were required in the majority of cases in this study (Table 1), illustrating that sequencing findings in isolation are usually insufficient to confidently confirm a diagnosis. Whether so-called incidental findings [49] are also reported will also effect this end cost [50].

Our experience of the application of WES in undiagnosed myopathies was favourable in comparison to sequential gene testing, but a detailed cost-benefit analysis of WES was out with the scope of this study. Studies addressing this question in other clinical scenarios are underway [51, 52]. In addition to the readily quantifiable expenses of confirmatory investigations or on-going genetic testing, any cost-benefit analysis of WES must consider the non-economic cost of remaining undiagnosed for several years and the psychological burden this places on individuals and families, in addition to the potential missed opportunities for diagnosis-specific interventions, involvement in clinical trials and ability to make informed reproductive choices [5, 11]. In rare diseases, where recruiting sufficient patients for clinic trials can be a barrier, each newly diagnosed person may contribute to advancement of translational research activities.

## Conclusions

Our results demonstrate the power of WES to provide diagnoses in patients with undiagnosed limb girdle weakness, including in singleton patients, with a clear advantage over sequential single gene testing in a cohort of clinically comparable patients. Earlier application of WES in the diagnostic pathway would be likely to substantially reduce time to diagnosis and may also reduce the costs incurred by ongoing investigations, as well as affording opportunities for detection of low level mosaicism and novel disease gene identification. As WES, or whole genome sequencing in the future, becomes increasingly commonplace the challenge will be to optimise its application in order to rationalise diagnostic investigations and provide a timely diagnosis for patients.

## Methods

### Evaluation of standard genetic testing

Sequential gene by gene testing and gene panels as were routinely available within the National Health Service (NHS) during the time period examined are referred to as “standard testing”. Details of the gene panels used are included in Additional file 1: Tables S1 and Additional file 2: Table S2. There was no LGMD gene panel available for diagnostic use through the NHS during the period studied. Data, including genetic tests performed, serum CK level, age of symptom onset, inheritance pattern and whether a muscle biopsy was performed, was collected for all undiagnosed patients attending the UK-LGMD clinic from 1st April 2013 to 31st March 2015. Six patients attending clinic in this time period underwent WES as part of this research study and therefore their data have been excluded from the analysis of standard genetic testing. All investigations were performed as part of standard clinical care.

### Patient selection for exome sequencing

Patients were selected from a database of those attending the UK-LGMD clinic between 2002 to present day. Individuals presenting with limb girdle weakness of a probably genetic aetiology who had been assessed by our neuromuscular specialist clinic at any time since 2002, and in whom genetic testing to date was negative, were selected. Priority was given to family structures likely to be most informative, for example those consistent with autosomal recessive inheritance or consanguineous families; however such families were relatively uncommon in this cohort of patients.

### Exome sequencing and bioinformatics

Affected individuals underwent WES; in families with apparent autosomal recessive inheritance two affected siblings were sequenced, and in those with autosomal dominant inheritance two affected family members were sequenced. If this did not yield a diagnosis additional affected or unaffected family members were subsequently sent for WES as appropriate.

WES in 30 individuals (20 affected, 10 unaffected) from 9 families was performed at de CODE genetics (Iceland) using Illumina Nextera Rapid Capture exome kit (37 Mb) and sequenced 90 nt paired-end on Illumina HiSeq2000 sequencer. WES in a further 104 (84 affected, 20 unaffected) individuals from 65 additional families was performed at the Genomics Platform at the Broad Institute of Harvard and MIT (Cambridge, MA, USA), with Agilent Sure-Select Human All Exon v2.0 (44 Mb) on Illumina HiSeqXs platform (Illumina, Inc. San Diego, CA). Sequence alignment, variant calling and functional annotation were performed with Burrows Wheel Aligner, Genomes Analysis Tool Kit and Variant Effect Predictor (VEP).

### Variant filtering and correlation with phenotype

Analysis of WES data was with proprietary software from deCODE genetics (Clinical Sequence Miner) and The Broad Institute (xBrowse) respectively. Variants were filtered to include those that were rare (MAF <0.01) in publically available reference datasets (ExAC and 1000 Genomes) and predicted to be moderately-severely damaging by VEP. Analysis was initially performed to identify variants in known muscle disease genes (www.musclegenetable.fr). Where a putatively pathogenic variant in a known gene was identified this was evaluated according to current guidelines including assessment of frequency, in silico predictions of pathogenicity, review of previous reports of patients with same variants, and published experimental work to review the effect of a variant on protein function [53, 54].

Putative genetic diagnoses were correlated with clinical findings including for example muscle MRI, clinical review of phenotype and assessment for extra-neuromuscular features as suggested by genotype. Where relevant and possible genetic diagnoses were correlated with histopathology phenotype, by identification of characteristic findings on muscle biopsy histopathology and immunoanalysis using antibodies typically using a standard dystrophy panel [55] as carried out by the diagnostic service provided by the Muscle Immunoanalysis Unit, which is part of the UK HSS LGMD referral service. Genetic findings, clinical features, muscle MRI and muscle biopsy results were then integrated at a multidisciplinary meeting to reach a consensus as to whether variants were likely to be disease causing.

In the absence of a positive diagnosis due to mutation in a known muscle disease gene additional affected or unaffected family members underwent WES and likely inheritance pattern and the scope of variant analysis was widened to include variants in novel genes.

## Additional files

Additional file 1: Table S1. Summary details of affected individuals included in WES cohort. (PDF 295 kb)

Additional file 2: Table S2. Summary details of affected individuals included in standard testing cohort. (PDF 272 kb)

Additional file 3: Figure. S1. Clinical details and muscle biopsy findings in families diagnosed with titinopathies. (PDF 340 kb)
