# Supplementary material for: Exome sequences versus sequential gene testing in the UK highly specialised Service for Limb Girdle Muscular Dystrophy
Source: Orphanet J Rare Dis. 2017 Sep 6;12:151. doi: 10.1186/s13023-017-0699-9 (PMC5588739; doi:10.1186/s13023-017-0699-9)
Supplement: Supplementary file 1 — Summary details of affected individuals included in WES cohort. (PDF 295 kb) [file 13023_2017_699_MOESM1_ESM.pdf]

family no	affected individual ID	Inheritance	Consanguineous	sex	age of onset*	CK	MB	previous genes screened (by any method)	no. genes tested	NGS gene panels tested	WES institute	no. affected individuals exomed	no. unaffected individuals exomed	undiagnosed / relevant disease gene if diagnosed
WES1	1	AR	no	m	adult	elevated <1000	yes	DMD, SM1, CAV3, ANO5, MADD, PYGM, CPT2	7		DeCode	2	0	undiagnosed
WES1	2			f	adult	elevated <1000	no							
WES2	1	AR	yes	f	childhood	elevated <1000	no	FKRP, CAPN3, SMN1, DYSF	4		DeCode	2	0	ZAK
WES2	2			m	childhood	elevated >1000	yes							
WES3	1	AR	no	m	childhood	normal	yes	FSHD1, LMNA, VCP	3		DeCode	2	0	undiagnosed
WES3	2			f	adult	normal	yes							
WES4	1	AR	yes	f	adult	elevated <1000	yes	FKRP, DMD, SCCA, SGCB, SGCD, SGDG, CAPN3	7		DeCode	3	1	undiagnosed
WES4	2			m	adult	not recorded	yes							
WES4	3			f	not recorded	not recorded	no							
WES5	1	AR	no	f	adult	normal	yes	BAG3, FKRP, FSHD1, DM2, CAPN3, SMN1, LMNA, DES, MYOT, VCP, FHL1	11		DeCode	3	0	TTN
WES5	2			f	teens	normal	yes							
WES5	3			f	teens	normal	yes							
WES6	1	AR	yes	m	childhood	elevated >1000	yes	LMNA, DOK7, FKRP ANO5	4		DeCode	1	5	undiagnosed
WES7	1	AR	no	m	adult	elevated >1000	yes	ANO5, DYSF, CAPN3, TTN*, LMNA, GNE	6		DeCode	2	2	undiagnosed
WES7	2			m	adult	elevated >1000	no							
WES8	1	sporadic	no	m	childhood	elevated <1000	yes	LMNA, FHL1, MSTN, PTRF	4		DeCode	1	2	undiagnosed
WES9	1	sporadic	no	f	childhood	elevated <1000	yes	BAG3, LMNA, SEPN1, FHL1, COL6A1, COL6A2, COL6A3	7		Broad	1	1	COL6A1
WES10	1	AR	no	m	teens	elevated <1000	yes	DM1, DM2, BAG3, DES, MYOT, CRYAB, FHL1	7		Broad	2	2	undiagnosed
WES10	2			f	adult	elevated <1000	yes							
WES11	1	AR	no	f	childhood	normal	yes	FKRP, SEPN1, LMNA, CAPN3, SMN1, POMT1, POMT2, POMGnT1, LARGE, FKTN, FHL1	11		Broad	2	2	undiagnosed
WES11	2			m	childhood	normal	no							
WES12	1	sporadic	no	m	childhood	elevated <1000	yes	LMNA, COL6A1, COL6A2, COL6A3, FHL1	5		Broad	1	0	undiagnosed
WES13	1	AD	no	m	childhood	not recorded	no	MYH2, MYH7, LMNA	3		Broad	4	0	PIEZO2
WES13	2			m	childhood	not recorded	no							
WES13	3			m	childhood	not recorded	no							
WES13	4			f	childhood	not recorded	no							
WES14	1	sporadic	yes	f	teens	elevated >1000	yes	ANO5, FKRP, CAPN3	3		Broad	1	0	undiagnosed
WES15	1	sporadic	no	m	childhood	elevated >1000	yes	LMNA, DYSF, ANO5, CAPN3, DMD, FKRP, AR, SCGA, SCGB, SCGG, SCGD, POMT1, POMT2, POMGnT1, LARGE, FKTN	16		Broad	1	2	undiagnosed
WES16	1	sporadic	no	m	adult	normal	yes	COL6A1, COL6A2, COL6A3, LMNA, FHL1, SEPN1, DM2, BAG3, CAPN3	9		Broad	1	2	undiagnosed
WES17	1	sporadic	no	f	adult	elevated >1000	yes	DNAJB6, TTN*, CAPN3, ANO5, FKRP, LMNA	6		Broad	1	2	undiagnosed
WES18	1	sporadic	no	f	childhood	not recorded	yes		0		Broad	1	0	TTN
WES19	1	sporadic	no	m	childhood	elevated >1000	yes	DMD, CAPN3, FKRP, LMNA, FHL1, TTN*, CCDC78, DYSF, DNAJB6, ANO5	10		Broad	1	0	TTN
WES20	1	sporadic	no	m	childhood	elevated >1000	yes	ANO5, POMT1, POMT2, POMGnT1, LARGE, FKTN, CAPN3, CAV3, FKRP, LMNA	10		Broad	1	2	undiagnosed
WES21	1	sporadic	no	m	childhood	elevated >1000	yes	DMD, LMNA, FHL1, CAPN3, SCGA, SCGB, SCGG, SCGD, COL6A1, COL6A2, COL6A3	11		Broad	1	0	undiagnosed
WES22	1	sporadic	no	f	adult	elevated <1000	yes	VCP, DES, MYOT,CRYAB, FSHD1	5		Broad	1	0	undiagnosed
WES23	1	AR	no	m	childhood	normal	yes	LMNA, FHL1, SEPN1, RYR1, DOK7, DNM2, ACTA1	7		DeCode	2	0	TTN
WES23	2			m	childhood	not recorded	no							
WES24	1	AR	yes	f	childhood	normal	yes	DOK7, RYR1, LMNA, RAPSN, CHRNG	5		Broad	1	2	undiagnosed
WES25	1	sporadic	no	m	adult	elevated >1000	yes	CAPN3, FHL1, ANO5, FSHD1, LMNA, FKRP, TPM3, FKRP, FKTN, POMT1, POMT2, POMGnT1, LARGE	13		Broad	1	0	GMPPB
WES26	1	AR	no	m	childhood	elevated <1000	yes	COL6A1, COL6A2, COL6A3, ANO5, CAPN3, LMNA, DMD	7		Broad	2	0	LAMA2
WES26	2			m	childhood	elevated <1000	no							
WES27	1	AR	no	m	teens	elevated >1000	yes	FKRP, DES, MYOT, ZASP, CRYAB, CAPN3, ANO5, SCGA, SCGB, SCGG, SCGD, DMD, DNAJB6, TTN*	14		Broad	1	0	undiagnosed
WES28	1	AR	no	f	adult	normal	yes	DNAJB6, TTN*, ZASP, CRYAB, MYOT, DES, SMN1, ANO5, CAPN3, DM2, DM1	11		Broad	2	0	undiagnosed
WES28	2			f	adult	normal	yes							

family no	affected individual ID	Inheritance	Consanguineous	sex	age of onset*	CK	MB	*previous genes screened (by any method)*	no. genes tested	NGS gene panels tested		no. affected individuals exomed	no. unaffected individuals exomed	undiagnosed / relevant disease gene if diagnosed
WES29	1	AR	no	f	childhood	elevated >1000	yes	*FKRP, COL6A1, COL6A2, COL6A3, LMNA, FHL1, SCGA, SCGB, SCGG, SCGD*	10		Broad	2	0	*TTN*
WES29	2			m	childhood	elevated >1000	no							
WES30	1	X-linked	no	m	childhood	elevated <1000	yes	*LMNA, SMA1, EMD, FHL1, DMD*	5		Broad	2	0	*MYH7*
WES30	2			m	childhood	not recorded	yes							
WES31	1	sporadic	no	m	adult	elevated <1000	yes	*DOK7, DM1, DM2, VCP, MYOT, DES, CRYAB, ZASP, DNAJB6, TTN**	10		Broad	1	0	*MEGF10*
WES32	1	sporadic	no	m	adult	elevated >1000	yes	*ANO5, DES, MYOT, FSHD1, FRKP, CAPN3, FHL1, DYSF, TTN*, SMN1, SGCA, SGCB, SGCG, SGCD*	14		Broad	1	0	*LAMA2*
WES33	1	sporadic	no	m	adult	elevated >1000	yes	*FKRP, VCP, ANO5, CAPN3, SCGA, SCGB, SCGG, SCGD, DNAJB6, TTN**	10		Broad	1	0	undiagnosed
WES34	1	sporadic	no	m	adult	elevated >1000	yes	*GNE, FSHD1, ANO5, VCP*	14		Broad	1	0	undiagnosed
WES35	1	sporadic	no	f	childhood	normal	yes	*MYH7 , SEPN1, RYR1, FHL1, DOK7, CFL2, ALG2*	7		Broad	1	0	undiagnosed
WES36	1	sporadic	no	f	adult	elevated >1000	yes	*DMD, DM2, DM1, PABN, DNAJB6, ZASP, DES, MYOT, CRYAB, VCP, TTN**	11		Broad	1	0	undiagnosed
WES37	1	sporadic	no	m	adult	elevated >1000	yes	*FHL1, LMNA, CAPN3, CAV3, FKRP, DM2, VCP, DES, CRYAB, ZASP, MYOT, TTN*, PABN1, ANO5, DM1, DNAJB6*	16		Broad	1	0	undiagnosed
WES38	1	sporadic	no	m	adult	elevated <1000	yes	*TTN*, ZASP, CRYAB, MYOT, DES, GNE, VCP, MYH7, FSHD1*	9		Broad	1	0	undiagnosed
WES39	1	sporadic	no	m	adult	elevated >1000	yes	*CAPN3, DYSF, GNE, DNAJB6, ANO5, TTN*, VCP*	7		Broad	1	0	*CAPN3*
WES40	1	sporadic	no	m	childhood	elevated >1000	yes	*DMD, DNAJB6, TTN*, FHL1, SCGA, SCGB, SCGG, SCGD, LAMA2, FKRP, POMT1, POMT2, POMGnT1, FKTN, LARGE, CAPN3*	16		Broad	1	0	undiagnosed
WES41	1	sporadic	no	m	childhood	elevated <1000	yes	*FSHD1, FSHD2, MYH7, TTN*, DNAJB6, FKRP, ANO5, VCP, DNM2, FHL1, DOK7, LMNA, CAPN3, DM2, DM1*	15		Broad	1	0	undiagnosed
WES42	1	sporadic	no	m	adult	elevated >1000	yes	*DMD, FKRP, FSHD1, LMNA, FLH1, CAPN3, ANO5, VCP, AR, TTN*, DNAJB6*	11		Broad	1	0	undiagnosed
WES43	1	sporadic	no	m	adult	elevated >1000	yes	*DES, MYOT, CRYAB, ZASP, TTN*, FKRP, CAPN3, ANO5, FSHD1, SMN1, VCP, LMNA*	12		Broad	1	0	undiagnosed
WES44	1	sporadic	no	m	adult	elevated >1000	yes	*CAPN3, DNAJB6, VCP, FKRP*	14		Broad	1	0	undiagnosed
WES45	1	sporadic	no	m	adult	elevated >1000	yes	*ANO5, VCP, DM1*	3		Broad	1	0	undiagnosed
WES46	1	X-linked recessiv	no	m	childhood	elevated <1000	yes	*ACTA1, TPM3, DNM2, MTM1,RYR1, TTN*, DNAJB6, FSHD1, LMNA*	9	hypertrophic cardiomyopathy (4 genes)	Broad	3	0	novel gene (unpublished)
WES46	2			f	childhood	not recorded	yes							
WES46	3			m	not recorded	elevated <1000	yes							
WES47	1	AD	no	f	childhood	elevated <1000	yes	*LMNA, DES, MYOT, FHL1, CRYAB, ZASP, TTN**	7		Broad	5	0	*COL6A1*
WES47	2			f	childhood	not recorded	not recorded							
WES47	3			f	childhood	not recorded	not recorded							
WES47	4			m	childhood	not recorded	not recorded							
WES47	5			m	childhood	not recorded	not recorded							
WES48	1	AD	no	m	adult	elevated <1000	yes	*DES, LMNA , ZASP, CRYAB, DNM2, DNAJB6, TTN*, FKRP, CAPN3, COL6, VCP, CAV3*	12		Broad	1	0	novel gene (unpublished)
WES49	1	AD	no	m	adult	elevated <1000	yes	*FLNC, FHL1, LMNA, SMN1*	13		Broad	3	0	*VCP*
WES49	2			f	adult	normal	no							
WES49	3			m	adult	not recorded	no							
WES50	1	sporadic	no	m	adult	elevated >1000	yes	DM2, *LMNA, FKRP, CAPN3, ANO5, DYSF, TTN*, DNAJB6*	8		Broad	1	0	undiagnosed
WES51	1	sporadic	no	m	adult	elevated <1000	yes	*VCP, MYOT, DES,CRYAB, FHL1, ANO5, ZASP, LMNA, TTN*, EMD,* FSHD1, DM2,	12		Broad	1	0	undiagnosed

family no	affected individual ID	Inheritance	Consanguineous	sex	age of onset*	CK	MB	previous genes screened (by any method)	no. genes tested	NGS gene panels tested		no. affected individuals exomed	no. unaffected individuals exomed	undiagnosed / relevant disease gene if diagnosed
WES52	1	AD	no	f	childhood	not recorded	no	LMNA, CAV3, MYOT, VCP, FHL1, DNAJB6, CRYAB, ZASP, CAPN3	9		Broad	2	0	COL6A3
WES52	2			f	childhood	normal	yes							
WES53	1	AD	no	m	childhood	elevated <1000	yes	LMNA, DES, MYOT, ZASP, CRYAB, BAG3, FHL1, DNAJB6, TTN*, MTM1	10		Broad	1	0	undiagnosed
WES54	1	sporadic	no	f	childhood	elevated >1000	yes	LARGE, FKTN, ANO5, CAPN3	13		Broad	1	2	undiagnosed
WES55	1	AR	no	m	childhood	elevated <1000	yes	FKRP, DM1, DOK7, FSHD	4		Broad	1	0	undiagnosed
WES56	1	sporadic	no	f	childhood	not recorded	yes	LMNA, COL6A1, COL6A2, COL6A3	4		Broad	1	0	STIM1
WES57	1	sporadic	no	m	childhood	elevated <1000	yes	LMNA, FHL1, EMD, DES, MYOT, CRYAB, ZASP	7		Broad	1	0	LMNA
WES58	1	sporadic	no	f	childhood	normal	yes	FKRP, DOK7, FHL1, MYH2, RYR1	5		Broad	1	0	undiagnosed
WES59	1	sporadic	no	m	childhood	elevated <1000	yes	ZASP, CRYAB, MYOT, DES, EMD, LMNA, FHL1, LMNA, VCP, TTN*	10		Broad	1	0	DNM2
WES60	1	sporadic	no	f	adult	elevated <1000	yes	VCP, MYH7, MYOM2, DNAJB6, VCP, TTN*, FSHD1, SMN1	8		Broad	1	0	undiagnosed
WES61	1	sporadic	no	m	adult	elevated >1000	yes	DNAJB6, TTN*, FHL1, BAG3, DES, MYOT, CRYAB, CAPN3, LMNA, EMD	10		Broad	1	0	TTN
WES62	1	sporadic	no	f	childhood	elevated <1000	yes	LMNA, FHL1, COL6A1, COL6A2, COL6A3, EMD, BAG3	7		Broad	1	0	STIM1
WES63	1	AD	no	f	adult	elevated <1000	yes	DES, MYOT, ZASP, CRYAB, DM2, VCP, FHL1, SEPN1, TTN*, DNAJB6, FSHD1, PABN1, MATR3	13		Broad	1	0	undiagnosed
WES64	1	sporadic	no	f	childhood	normal	yes	LMNA, FKRP, CAPN3, SMN, FHL1, VCP	6		Broad	1	0	undiagnosed
WES65	1	AD	no	m	childhood	elevated >1000	yes	LMNA, FHL1, COL6A1, COL6A2, COL6A3	5		Broad	2	0	CAV3
WES65	2			f	childhood	elevated <1000	yes							
WES66	1	sporadic	no	f	childhood	normal	yes	RYR1, LMNA, SEPN1	3		Broad	1	0	undiagnosed
WES67	1	sporadic	no	f	adult	normal	yes	CRYAB, TTN*	12		Broad	1	0	MTM1
WES68	1	sporadic	no	m	adult	elevated >1000	yes	CAPN3, DYSF, DNAJB6, FKRP, TTN*, ANO5, MYOT	7		Broad	1	0	undiagnosed
WES69	1	sporadic	no	m	childhood	elevated <1000	yes	EMD, FHL1, LMNA, SMN1, RYR1, VCP	6		Broad	1	2	undiagnosed
WES70	1	sporadic	no	m	teens	elevated >1000	yes	ANO5, FKRP, CAV3, CAPN3	4		Broad	1	2	undiagnosed
WES71	1	sporadic	no	m	adult	elevated >1000	yes	DMD, CAV3, ANO5, FKRP, FSHD1	5		Broad	1	0	DYSF
WES72	1	sporadic	yes	m	teens	elevated <1000	yes	DM2, FKRP, ANO5, CAPN3, RYR1, LMNA	6		Broad	1	0	undiagnosed
WES73	1	sporadic	no	m	childhood	elevated >1000	yes	DOK7, COLQ, RAPSN, TPM3, ACTA1	5		Broad	1	0	undiagnosed
WES74	1	AR	no	f	childhood	normal	yes	CHAT, RAPSYN, DOK7	3		Broad	1	0	undiagnosed
WES75	1	AR	yes	m	childhood	elevated >1000	yes	DMD, FKRP, SGCA, SGCB, SGCG, SGCD	6		DeCode	2	0	SGCG
WES75	2			m	childhood	elevated >1000	yes							SGCG

Note: * testing of TTN was limited to sequencing of mutation hotspots associated with Herediatry Myopathy with Early Respiratory Failure and no whole gene sequencing was performed. Childhood onset denotes onset from birth to teenage years. DM1 or DM2 refer to genetic testing for Myotonic dytrophy type 1 or 2 respectively; FHSD1 or FSHD2 refers to genetic testing for Fascioscapulohumeral Muscular Dystrophy type 1 or 2.
