# Supplementary material for: Exome sequences versus sequential gene testing in the UK highly specialised Service for Limb Girdle Muscular Dystrophy
Source: Orphanet J Rare Dis. 2017 Sep 6;12:151. doi: 10.1186/s13023-017-0699-9 (PMC5588739; doi:10.1186/s13023-017-0699-9)
Supplement: Supplementary file 2 — Summary details of affected individuals included in standard testing cohort. (PDF 272 kb) [file 13023_2017_699_MOESM2_ESM.pdf]

family no	individual ID	Inheritance	Consanguineous	sex	age of onset	CK	MB	genes screened (by any method)	number of genes tested	NGS gene panels tested	diagnosis
STND1	1	sporadic	no	m	adult	not recorded	no	*LMNA, FHL1*	1		undiagnosed
STND2	1	AD	no	f	childhood	normal	yes	*LMNA, SMN1, SEPN1, RYR1, DES, CRYAB, MYOT, FHL1, MYH7*	9		*MHY7* related myopathy
STND3	1	sporadic	no	m	adult	elevated <1000	yes	*FKRP, CAPN3, LMNA*	13		undiagnosed
STND4	1	sporadic	no	m	adult	elevated <1000	yes	*FHL1, LMNA, STIM1*	3		*STIM1*
STND5	1	sporadic	no	m	childhood	elevated >1000	yes	*ANO5, CAPN3, FKRP, LMNA, FSHD1 FHL1, TCAP*	7		LGMD2G due to TCAP mutation
STND6	1	sporadic	no	m	adult	elevated >1000	yes	*CAPN3, ANO5, FKRP, DYSF, MYOT, TTN*, VCP*	7		undiagnosed
STND7	1	sporadic	no	f	adult	elevated <1000	yes	*FHL1, GNE, DES, MYOT*	4		Desminopathy
STND8	1	sporadic	no	f	childhood	normal	yes	*LMNA, DES, TTN*, CRYAB*	4		undiagnosed
STND9	1	sporadic	no	m	adult	normal	yes	*POLG, PE01, RRM2B, MYH3, DNM2*	5	congenital myopathies (22 genes)	undiagnosed
STND10	1	sporadic	yes	m	childhood	elevated >1000	yes	*MYH7, ANO5, FKRP, CAPN3*	5		undiagnosed
STND11	1	sporadic	no	f	childhood	elevated >1000	yes	*ANO5, DMD, SGCA, SGCB, SGCD, SGCG,*	6		undiagnosed
STND12	1	sporadic	no	f	adult	elevated >1000	yes	*FKRP, CAPN3, ANO5, ORAI1, STIM1*	5	rhabdomyolysis research panel	undiagnosed
STND13	1	sporadic	no	m	adult	elevated >1000	yes	*ANO5, FKRP, CAPN3, MYOT, DES, CRYAB, ZASP, TTN*, VCP, DNAJB6*	10		undiagnosed
STND14	1	sporadic	no	m	adult	elevated <1000	yes	*DES, GNE, DM1, DM2, MYH7, MYOT, CRYAB, DES, ZASP, TTN*, FSHD1*	11		undiagnosed
STND15	1	AD	no	m	childhood	normal	yes	*MYH7, DMD, FKRP, LMNA, FHL1, VCP, ZASP, CRYAB, MYOT, DES, ANO5, TTN**	12		undiagnosed
STND16	1	sporadic	no	f	adult	normal	yes	*LMNA, RYR1*	2	congenital myopathies (22 genes)	undiagnosed
STND17	1	sporadic	no	m	adult	elevated >1000	yes	*ANO5, FHL1, FKRP, TTN*, GNE, CAPN3, DYSF*	7		undiagnosed
STND18	1	AR	no	f	childhood	elevated <1000	yes	*CAPN3, LMNA, TTN*, COL6A1, COL6A2, COL6A3*	6		collagen VI related disease
STND18	2	AR	no	m	childhood	elevated <1000	yes				collagen VI related disease
STND19	1	sporadic	no	f	adult	not recorded	yes	*LMNA, DES, MYOT, CRYAB, ZASP, TTN* MYH7*	13		undiagnosed
STND20	1	sporadic	no	m	childhood	normal	yes	*CAPN3, LMNA, FHL1, EMD*	4		undiagnosed
STND21	1	sporadic	no	m	adult	normal	yes	*FSHD1, VCP, FHL1, FSHD2*	4		FSHD2
STND22	1	AR	yes	f	childhood	not recorded	yes	*COL6A1, COL6A2, COL6A3*	3		collagen VI related disease
STND22	2	AR	yes	f	childhood	elevated <1000	yes				collagen VI related disease
STND23	1	AD	no	m	childhood	normal	yes	*MYH7, LMNA, DES, MYOT, CRYAB, ZASP, TTN*, VCP*	8		undiagnosed
STND24	1	sporadic	no	f	adult	normal	yes	*MYH7, MYOT, CRYAB, VCP, FHL1, ZASP, PMP22*	7	peripheral neuropathies (56 genes)	CMT2A due to *MFN2* mutation
STND25	1	sporadic	no	m	childhood	normal	yes	*LMNA, CAPN3, DM2, FSHD1, ANO5, DYSF*	6		undiagnosed
STND26	1	sporadic	no	m	adult	not recorded	yes	*FSHD1 LMNA, VCP*	3		*VCP* myopathy
STND27	1	sporadic	no	m	adult	elevated <1000	yes	*DM2, CAPN3, FSHD1, DMD*	4		undiagnosed
STND28	1	sporadic	no	f	adult	elevated >1000	yes	*DM1, TTN*, FSHD1 LMNA, DNM2, DES, ZASP, CRYAB, MYOT*	9		undiagnosed
STND29	1	sporadic	no	m	adult	normal	yes	*McArdles, TTN*, CAPN3*	3		undiagnosed
STND30	1	sporadic	no	f	adult	normal	yes	*LMNA*	1		undiagnosed
STND31	1	sporadic	no	m	adult	elevated >1000	yes	*DMD, FKRP, ANO5*	3	hypertrophic cardiomyopathy (4 genes)	undiagnosed
STND32	1	sporadic	no	m	childhood	not recorded	no	*CHRNE, RAPSN, DOK7, CHRNA1, CHRND, CHRNB1*	6		*CHRNB1* -slow channel myasthenic syndrome
STND33	1	sporadic	no	m	adult	elevated >1000	yes	*CAV3, DM2, FKRP, ANO5, CAPN3*	6		undiagnosed
STND34	1	AR	no	f	childhood	normal	yes	*DM1, DM2, FSHD1, FSHD2, SMN1*	5		SMA
STND35	1	sporadic	no	m	childhood	elevated <1000	yes	*FSHD1, CAPN3, FHL1, FSHD2, LMNA, EMD, ANO5*	7		undiagnosed
STND36	1	sporadic	no	f	childhood	elevated >1000	yes	*CAPN3*	1		LGMD2A due to *CAPN3* mutation
STND37	1	sporadic	no	f	adult	elevated <1000	yes	*DMD, ANO5, CAPN3*	3		LGMD2A due to *CAPN3* mutation
STND38	1	AD	no	m	childhood	not recorded	not recorde	*FSHD1*	1		FSHD1
STND39	1	sporadic	no	f	adult	elevated >1000	yes	*TTN*, MYH7, RYR1, DM1*	4		undiagnosed
STND40	1	sporadic	no	f	adult	elevated <1000	yes	*FKRP, CAPN3, POMT1, POMT2, LARGE, FKTN, POMGnT1, DES, LMNA, ANO5, TTN*, MYOT, CRYAB, ZASP, FHL1*	15		*FHL1*
STND41	1	sporadic	no	m	childhood	elevated <1000	yes	*FSHD1, CAV3, DMD, PYGM*	4		undiagnosed

family no	individual ID	Inheritance	Consanguineous	sex	age of onset	CK	MB	genes screened (by any method)	number of genes tested	NGS gene panels tested	diagnosis
STND42	1	sporadic	no	m	childhood	normal	yes	*DOK7, RAPSN*	2	ataxias research panel	undiagnosed
STND43	1	sporadic	no	f	adult	elevated >1000	yes	*RYR1*	1		*RYR1* related myopathy
STND44	1	sporadic	no	f	adult	normal	yes	*DES, MYOT, CRYAB, ZASP, TTN**, MYH7, VCP, PABN1, MATR3*	9		undiagnosed
STND45	1	sporadic	no	f	childhood	elevated >1000	yes	*LMNA, FSHD1, DMD, SMN1*	4	congenital muscular dystrophies (31 genes)	undiagnosed
STND46	1	sporadic	no	m	childhood	normal	yes	*DES, MYOT, CRYAB, ZASP, LMNA, RYR1*	6		undiagnosed
STND47	1	sporadic	no	m	adult	elevated >1000	yes	*DMD, FKRP, CAPN3, DYSF*	4		Dysferlinopathy
STND48	1	AR	no	f	adult	elevated >1000	no	*ANO5, DYSF*	2		Dysferlinopathy
STND48	2	AR	no	f	adult	elevated >1000	yes				LGMD2B due to *DYSF* mutations
STND48	3	AR	no	m	adult	elevated >1000	no				LGMD2B due to *DYSF* mutations
STND49	1	sporadic	no	m	adult	elevated <1000	yes	*TTN exon 334, CRYAB, MYOT, DES, TCAP, ZASP, GNE*	7		undiagnosed
STND50	1	sporadic	no	f	adult	elevated <1000	yes	*RYR1, VCP, FHL1, LMNA, DM2, CFL2, MYH7, MTM1, CFL2, RYR1, FSHD1 DM2*	12		undiagnosed
STND51	1	X-linked	no	m	childhood	elevated >1000	yes	*SEPN1, LMNA, RYR1, FHL1, FKRP, MYH7*	6		undiagnosed
STND52	1	sporadic	no	m	childhood	normal	yes	*LMNA, COL6A1, COL6A2, COL6A3,*	4		undiagnosed
STND53	1	sporadic	no	f	childhood	elevated <1000	yes	*SGCA, SGCB, SGCD, SGCG, CAPN3, DM2, TCAP, ANO5, FKRP, GNE*	10		undiagnosed
STND54	1	sporadic	no	m	adult	elevated >1000	yes	*TTN HMEF c.m., CAPN3, DYSF, MYOT, FKRP, ANO5*	6		undiagnosed
STND55	1	AD	no	f	adult	not recorded	yes	*LMNA, COL6A1, COL6A2, COL6A3,*	4		undiagnosed
STND56	1	sporadic	no	f	childhood	not recorded	no	*LMNA, FHL1*	2		undiagnosed
STND57	1	sporadic	no	f	adult	elevated >1000	yes	*ANO5, FSHD1, DMD*	3		DMD manifesting carrier
STND58	1	sporadic	no	m	adult	elevated >1000	yes	*CAPN3, ANO5, FKRP, MYOT, ZASP, DES, TTN HMERRF, c.m., DYSF*	8		undiagnosed
STND59	1	sporadic	no	f	adult	elevated <1000	yes	*FSHD1*	1	congenital myopathies (22 genes)	undiagnosed
STND60	1	AD	no	f	childhood	normal	no	*m.3243A>G MELAS mutation, GCH1*	2		GCH1 deletion dystonia
STND60	2	AD	no	f	childhood	normal	no				GCH1 deletion dystonia
STND60	3	AD	no	f	childhood	normal	no				GCH1 deletion dystonia
STND61	1	sporadic	no	f	adult	elevated >1000	yes	*BAG3, FKRP, ANO5, CAPN3*	4		undiagnosed
STND62	1	sporadic	no	f	adult	elevated >1000	yes	*ZASP, DES, MYOT, CRYAB, TTN**, LMNA, DM2*	7		undiagnosed
STND63	1	sporadic	yes	m	adult	elevated >1000	no	*ANO5, DYSF*	2		Dysferlinopathy
STND64	1	sporadic	no	m	adult	elevated <1000	yes	*FSHD1 NEB, MYH7, CAPN3*	4	peripheral neuropathies (56 genes), titin	undiagnosed
STND65	1	sporadic	no	m	adult	elevated <1000	yes	*DES, MYOT, ZASP, CRYAB*	4		Myotilinopathy
STND66	1	sporadic	no	f	adult	elevated >1000	yes	*DM2, ANO5, FKRP, CAPN3*	4		undiagnosed
STND67	1	sporadic	no	m	childhood	elevated <1000	no	*COL6A1, COL6A2, COL6A3*	3		Collagen VI related disease
STND68	1	AR	yes	m	childhood	normal	yes	*CYP7B1*	1	muscle channelopathies (4 genes)	HSP type 5A
STND69	1	sporadic	no	f	adult	elevated <1000	yes	*GAA, DES, ZASP, MYOT, CRYAB, TTN**, VCP*	7	peripheral neuropathies (56 genes)	undiagnosed
STND70	1	AD	no	m	adult	normal	yes	*TTN**, DES, CRYAB, ZASP, MYOT, VCP, LMNA, FSHD1, GNE*	9		undiagnosed
STND71	1	sporadic	no	m	adult	elevated <1000	yes	*TTN**, DES, CRYAB, ZASP, MYOT, LMNA*	6	titin	undiagnosed
STND72	1	sporadic	no	m	adult	normal	yes	*GNE, DES, MYOT, ZASP, CRYAB, TTN**, MYH7, VCP,*	8		undiagnosed
STND73	1	sporadic	no	m	adult	normal	no	*DMD, FKRP*	2		undiagnosed
STND74	1	AD	no	m	adult	elevated >1000	yes	*ZASP, CRYAB, DES, MYOT, TTN**	5		Myotilinopathy
STND75	1	sporadic	no	m	childhood	normal	yes	*TTN**, SEPN1, LMNA, EMD, FHL1, RYR1*	6	titin	undiagnosed
STND76	1	sporadic	no	m	childhood	normal	yes	*LMNA, SMN1, FSHD1*	3		undiagnosed
STND77	1	sporadic	no	f	childhood	elevated >1000	yes	*CAPN3*	1		LGMD2A due to *CAPN3* mutation
STND77	1	AR	no	m	childhood	normal	yes	*LMNA, MYH7*	2	congenital myopathies (22 genes)	undiagnosed
STND77	2	AR	no	m	childhood	normal	yes				undiagnosed
STND79	1	sporadic	no	m	childhood	normal	no	*LMNA, EMD, FBN1*	3		undiagnosed

family no	individual ID	Inheritance	Consanguineous	sex	age of onset	CK	MB	genes screened (by any method)	number of genes tested	NGS gene panels tested	diagnosis
STND80	1	AD	no	m	adult	elevated <1000	yes	*LMNA, DNAJB6, FLNC, ZASP, CRYAB, DES, MYOT, TTN*, COLA1, COL6A2, COL6A3*	11		undiagnosed
STND81	1	sporadic	no	m	adult	elevated <1000	yes	*DM2, TTN*, GNE*	1		*GNE* myopathy
STND82	1	sporadic	no	m	adult	elevated >1000	yes	*ANO5, CAPN3, FSHD1 CAV3, DM2*	5	rhabdomyolysis research panel	undiagnosed
STND83	1	AR	no	m	adult	elevated >1000	yes	*FKRP, CAPN3, DYSF, ANO5, FSHD1 STIM1, ORAI1, DM2*	8		undiagnosed
STND84	1	sporadic	yes	m	adult	elevated >1000	yes	*ANO5, VCP, GNE*	3		*GNE* myopathy

Note: * testing of TTN was limited to sequencing of mutation hotspots associated with Herediatry Myopathy with Early Respiratory Failure and no whole gene sequencing was performed. Childhood onset denotes onset from birth to teenage years. DM1 or DM2 refer to genetic testing for Myotonic dytrophy type 1 or 2 respectively; FHSD1 or FSHD2 refers to genetic testing for Fascioscapulohumeral Muscular Dystrophy type 1 or 2.
