# Supplementary material for: Exome sequences versus sequential gene testing in the UK highly specialised Service for Limb Girdle Muscular Dystrophy
Source: Orphanet J Rare Dis. 2017 Sep 6;12:151. doi: 10.1186/s13023-017-0699-9 (PMC5588739; doi:10.1186/s13023-017-0699-9)
Supplement: Supplementary file 3 — Clinical details and muscle biopsy findings in families diagnosed with titinopathies. (PDF 340 kb) [file 13023_2017_699_MOESM3_ESM.pdf]

Supplemental data for families diagnosed with titinopathies

Details of families WES19, 29 and 61 have been reported separately (1).

WES5

Family WES5 comprised two affected siblings (patients 1 and 2) whose mother died in her 30s from an unknown cause and an affected maternal cousin (patient 3). Clinical phenotype was of onset in early adulthood or teens of myopathy with proximal limb weakness and severe weakness of plantar flexion. MRI of the lower limbs in patient WES5: patient 2 showed replacement of muscle by fatty tissue in the posterior compartment of the thighs, and in the lower limbs of the tibialis anterior. Muscle biopsy was reviewed in light of identification of titin mutations, and showed rimmed vacuoles, eosinophilic inclusions and myotillin inclusions in WES5: patient 1 (see figure).

WES18

Family WES18 comprised a single affected individual with congenital hypotonia and delayed motor milestones, dilated cardiomyopathy and respiratory failure. Muscle MRI was normal. Muscle biopsy showed variation in fibre size, centrally located nuclei and myotilin accumulation with protein aggregates similar that those previously reported in Hereditary Myopathy with Early Respiratory Failure (HMERF) (2) (see figure).

The affected individual in family WES18 has been included in a manuscript describing a cohort of congenital titinopathy patients (patient D179253), currently in preparation.

WES23

Family WES23 comprised two brothers with onset in childhood of proximal limb weakness. In addition WES23: patient 1 had axial and facial weakness, rigid spine, scoliosis, mitral valve prolapse, cardiomyopathy and respiratory failure. WES29: patient 2 had respiratory insufficiency. Muscle MRI in WES29: patient 1 showed mild involvement of the semitendinosis, and in WES29: patient 2 showed mild non-specific changes. Muscle biopsy in WES23: patient 1 was reported as showing

multiminicores (not visible on H&E shown here), H&E shows basophilic cytoplasmic areas (arrow), predominance of type 1 fibres as shown with slow myosin, and groups of fibres with un-specific cytoplasmic accumulation of myofibrillar proteins (myotilin shown).

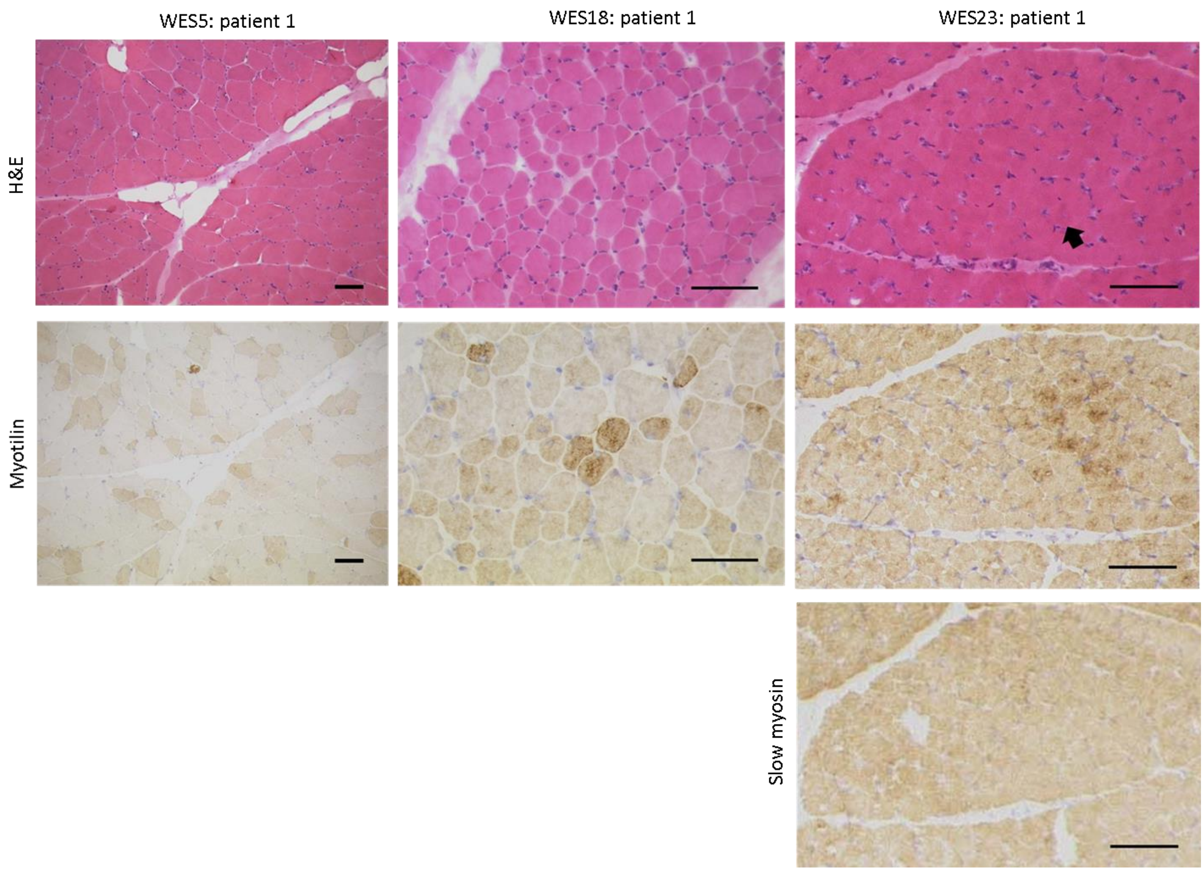

Scale bars 100µm

References

1. Harris E, Topf A, Vihola A, Evila A, Barresi R, Hudson J, et al. A 'second truncation' in TTN causes early onset recessive muscular dystrophy. Neuromuscular disorders : NMD. 2017.

2. Pfeffer G, Barresi R, Wilson IJ, Hardy SA, Griffin H, Hudson J, et al. Titin founder mutation is a common cause of myofibrillar myopathy with early respiratory failure. Journal of neurology, neurosurgery, and psychiatry. 2014;85(3):331-8.
